# Development of a diarrhoea severity scoring scale in a passive health facility-based surveillance system

**DOI:** 10.1371/journal.pone.0272981

**Published:** 2022-08-15

**Authors:** Denise T. St Jean, Obvious N. Chilyabanyama, Samuel Bosomprah, Mah Asombang, Rachel M. Velu, Mwelwa Chibuye, Fiona Mureithi, Nsofwa Sukwa, Masuzyo Chirwa, Prudence Mokha, Roma Chilengi, Michelo Simuyandi

**Affiliations:** 1 Department of Epidemiology, Gillings School of Global Public Health, University of North Carolina at Chapel Hill, Chapel Hill, North Carolina, United States of America; 2 Centre for Infectious Diseases Research in Zambia, Lusaka, Zambia; 3 Department of Biostatistics, School of Public Health, University of Ghana, Ghana, Accra; Federal University of Sergipe, BRAZIL

## Abstract

**Background:**

Diarrhoeal disease remains a leading cause of death among children mostly in low and middle-income countries. Factors contributing to disease severity are complex and there is currently no consensus on a scoring tool for use in community-based studies.

**Methods:**

Data were collected during a passive surveillance system in an outpatient health facility in Lusaka, Zambia from March 2019 to July 2019. Diarrhea episodes were assessed for severity using an in-house severity scoring tool (CIDRZ) and previously published scores (Vesikari, Clark, CODA, and DHAKA). The CIDRZ score was constructed using fieldworker-reported clinical signs and exploratory factor analysis. We used precision-recall curves measuring severe diarrhoea (i.e., requiring intravenous rehydration or referred for hospital admission) to determine the best performing scores. Then, we used Cronbach’s alpha to assess the scale’s internal consistency. Finally, we used Cohen’s kappa to assess agreement between the scores.

**Results:**

Of 110 diarrhea episodes, 3 (3%) required intravenous rehydration or were referred for hospital admission. The precision-recall area under the curve of each score as a predictor of severe diarrhoea requiring intravenous rehydration or hospital admission was 0.26 for Vesikari, 0.18 for CODA, 0.24 for Clark, 0.59 for DHAKA, and 0.59 for CIDRZ. The CIDRZ scale had substantial reliability and performed similarly to the DHAKA score.

**Conclusions:**

Diarrhoea severity scores focused on characteristics specific to dehydration status may better predict severe diarrhea among children in Lusaka. Aetiology-specific scoring tools may not be appropriate for use in community healthcare settings. Validation studies for the CIDRZ score in diverse settings and with larger sample sizes are warranted.

## Introduction

Although the burden of diarrhoeal disease has steadily declined in recent years, diarrhoea still accounts for a large proportion of global childhood morbidity and mortality, particularly in low-and-middle-income countries (LMICs) [1–3]. In healthcare settings, the severity of diarrhoeal episodes is a factor of interest due to its implications for health outcomes, recommended treatment, and clinical research applications. Moderate-to-severe diarrhoeal disease is associated with a higher risk of acute morbidity, later morbidity such as malnutrition, and mortality due to dehydration compared to mild-to-moderate diarrhoeal episodes [4–6]. Since mortality is more likely in cases of severe diarrhoea, accurate clinical assessment of severe diarrhoea is critical to preventing severe dehydration and its complications.

Several diarrhoeal disease severity scoring systems have been developed for uses ranging from community-based active surveillance [7] to characterization of aetiologic-specific diarrhoea [8–11]. Factors that contribute to the severity of diarrhoeal diseases in children are complex, including child demographics, virulence of aetiologic agents [12], socio-economic status, co-morbidities [13], environmental characteristics [14], and behavioural factors [15]. However, there is currently no consensus on which severity scoring tool to use to define severity of non-aetiologic specific diarrhoea, that can accurately predict poor health outcomes in resource limited settings [16]. While the Vesikari [8] and Clark [11] scores are well-validated in assessing rotavirus severity and the COmmunity DiarrhoeA (CODA) score [17] is considered to be predictive of long-term health outcomes in a community context, there may be important differences in non-aetiologic disease assessment in outpatient settings. And though the World Health Organization (WHO) Integrated Management of Childhood Illness (IMCI) guidelines [18] and Dehydration: Assessing Kids Accurately (DHAKA) score [17] are designed as clinical classification rubrics for dehydration severity, incorporating diarrhoea-specific characteristics may improve the likelihood that the correct treatment is assigned.

Given the public health importance of diarrhoeal disease as global contributor to childhood morbidity and mortality, it is essential to have tools that accurately characterize disease severity. Using data from a passive health facility-based diarrhoea surveillance system, we developed a diarrhoeal severity scale for the Centre for Infectious Disease Research in Zambia (CIDRZ), combining variables from the existing major scores to predict severe diarrhoea. We assessed the scale in an outpatient healthcare setting, alongside four previously published scores, against diarrhoeal episodes requiring hospitalisation or intravenous (IV) rehydration.

## Methods

### Data collection

Data were collected during a passive surveillance system for diarrhoea among children under five years conducted at Chainda South health facility from March 2019 to July 2019. Children under five experiencing diarrhoea, based on primary caregiver report at presentation to the health facility, and whose caregiver provided informed consent were included in the study. Children were eligible even if diarrhea was not the primary presenting complaint. The facility is located in a peri-urban settlement area of Lusaka and serves a community with a catchment population of over 25,000 [19]. All eligible children whose primary caregiver provided informed consent were enrolled. Chainda South provides health care services ranging from mother and child health, outpatient, immunization services, and more for populations of Kalikiliki compound, Mtendere East, parts of Ibex Hill and Salama Park. Data was collected from the child’s mother or primary caregivers on child demographics, HIV (human immunodeficiency virus) status, geographic area of residence, and immunization history. Further, clinical disease symptoms and prescribed treatment were noted.

### Ethics statement

The study was approved by the University of Zambia Biomedical Ethics Committee (UNZABREC) and was authorized by the National Health Research Authority (NHRA). Written informed consent from all participant’s caregivers was obtained prior to any study related activities.

### Severity scores for comparison

Four existing major scoring systems with modifications based on data availability described in Table 1. In summary, the Vesikari score [8] was modified based on available data: substitution of percentage of dehydration with categorical measures of some or severe dehydration, exclusion of duration of vomiting, and exclusion of treatment. Next, the Clark score [11] was modified based on collected data: replacement of rectal temperature with axillary temperature, substitution of behavioral symptoms irritable = 1, lethargic/listless = 2, and seizures = 3 with restless/ irritable = 1 and lethargic = 2. exclusion of duration of vomiting, and exclusion of duration of behavioural signs. Finally, the CODA score [7] was modified as follows: duration of anorexia was replaced with presence or absence of anorexia, duration of fever was replaced with presence or absence of fever, and duration of vomiting was replaced by the maximum number of vomiting per day. No modifications were made to the DHAKA score [17].

**Table 1:** Vesikari, Clark, CODA, DHAKA, and CIDRZ scores.

Score component	Modified Vesikari^a^	Modified Clark^b^	Modified CODA^c^	DHAKA	CIDRZ	Scoring
Duration of diarrhoea (in days)	1–4	1–4	--	--	--	1
	5	5–7	--	--	--	2
	≥6	≥8	--	--	--	3
Max number of loose stools per day	1–3	2–4	4–5	--	--	1
	4–5	5–7	6–7	--	--	2
	≥6	≥8	≥8	--	--	3
Max number of times vomiting per day	1	1–3	1–2	--	2–3	1
	2–4	4–6	3–4	--	4–5	2
	≥5	≥7	≥5	--	≥6	3
Fever	--	--	≥37.5°C	--	--	1
Confirmed temperature	37.1–38.4°C	38.0–38.2°C	--	--	--	1
	38.5–38.9°C	38.3–38.7°C	--	--	--	2
	≥39.0°C	≥38.8°C	--	--	--	3
Behavioural signs	--	Normal	--	--	--	0
	--	Restless/ irritable	--	--	--	1
	--	Lethargic	--	Restless/ irritable	Restless/ irritable	2
	--	--	--	--	Lethargic	3
	--	--	--	Lethargic	--	4
Anorexia	--	--	Present	--	--	1
Hydration status	No dehydration	--	No dehydration	--	--	0
	Some dehydration	--	Some dehydration	--	--	2
	Severe dehydration	--	Severe dehydration	--	--	3
Skin pinch	--	--	--	Normal	Normal	0
	--	--	--	Slow	Slow	2
	--	--	--	--	Very slow	3
	--	--	--	Very slow	--	4
Tears	--	--	--	Decreased	Decreased	1
	--	--	--	Absent	Absent	2
Respirations	--	--	--	Normal	Normal	0
	--	--	--	Deep	Deep	2
**Max number of points**	**15**	**14**	**11**	**12**	**13**	

^a^ Percentage of dehydration was replaced with categorical measures of some or severe dehydration; duration of vomiting was excluded; treatment was excluded as it was considered a proxy for outcome.

^b^ Duration of vomiting and duration of behavioural signs were excluded; behavioural signs were categorized as restless/ irritable = 1 and lethargic = 2; rectal temperature was replaced with axillary temperature.

^c^ Duration of anorexia and fever were replaced with presence or absence of these symptoms; duration of vomiting was replaced by the maximum number of vomiting per day.

### Item and scale development for the CIDRZ scale

The item (or factor) pool were identified using characteristics from the existing major scoring systems described above. A total of 11 factors were generated and consolidated: duration of diarrhoea, the maximum number of loose stools per day, maximum number of vomiting per day among children experiencing vomiting, fever, axillary temperature, behavioural signs, anorexia, hydration status, skin pinch, tears, and respirations. Duration of diarrhoea, the maximum number of loose stools per day, and the maximum number of vomiting per day were continuous variables reported by the primary caregiver and collected at presentation. Fever was a binary variable defined as an axillary temperature of 37.5°C or greater. Because the proportion of missing data for these continuous covariates was small, we imputed the median values of each variable for individuals with missing data. Anorexia, or ability to feed, was a dichotomous variable reported by the primary caregiver. Behavioural signs, hydration status, skin pinch, tears, and respirations were categorical variables relying on evaluation by a clinician. Hydration status was assessed per the WHO ICMI definition [18]. Hospitalisation or diarrhoea requiring IV rehydration, which usually indicates severe dehydration, were considered proxies for severe diarrhoea, consistent with previous literature [16,20,21]. Diarrhoea treated promptly in the home to prevent and treat dehydration rarely leads to death; therefore, utilizing diarrhoea hospitalisation and IV rehydration as outcomes allows us to focus on more severe episodes more likely to lead to more serious outcomes.

We used exploratory factor analysis (EFA) to extract factors and assess their contribution to severity using rotated factor loadings [22,23]. Items with orthogonal rotated factor loadings < 0.40 were excluded from the scale [24]. Also, items with cross-loadings or that appear to not load uniquely on individual factors were deleted. The number of factors extracted was determined based on eigenvalues > 1. We used Cronbach’s alpha to assess the internal consistency of the scale items [24,25]. An alpha coefficient of ≥ 0.70 was considered an acceptable threshold for reliability.

### Scale evaluation

Receiver operating characteristic (ROC) curves, precision-recall curves, and area under the precision-recall curve (AUC) were used to compare each score with the outcome, severe diarrhoea (i.e. requiring IV rehydration or referred for hospital admission). First, an ROC curve analysis was performed to select the cut-off for moderate-to-severe diarrhoea for each score. The score that maximized the sum of sensitivity and specificity was selected as the cut-off. In a precision-recall curve analysis, we then identified scores that maximized the AUC, representing both high precision (i.e., a lower false positive rate) and high recall (i.e., a lower false negative rate). Finally, Cohen’s kappa (k) was calculated to assess agreement in classifying severe diarrhoea between the CIDRZ score and each of the existing diarrhoea severity score; k≥0.60 was considered substantial agreement. All analyses were performed using Stata version 15 (StataCorp, College Station, TX, USA).

## Results

### Background characteristics of participants

A total of 120 children with acute diarrhoeal illness presented to the clinic. Four children were excluded due to missing data on the visit outcome while six children were excluded due to missing data on clinical symptoms of disease severity giving a total of 110 children for analysis. All study participants resided in the Lusaka urban district, with almost all children (94%, n = 102) residing in the Mtendere East neighbourhood. Approximately 57% of all children were male and the median age of children presenting with diarrhoea was 14 months. Of 94 children with a known status, 93% (n = 87) were HIV-negative (Table 2).

**Table 2:** Characteristics of study population (n = 110).

Characteristics	n (%) or median (IQR)
Age in months *(n = 109)*	14 (10, 23)
Male sex	63 (57)
Neighbourhood (*n = 108)*	
Mtendere East	102 (94)
Ibex	4 (4)
Kalikiliki	2 (2)
HIV-positive *(n = 94)*	7 (7)
Weight before treatment (in kilograms)	10 (8, 11)

### Clinical characteristics of diarrhoeal episodes

Of the 110 children presenting to the clinic, all experienced diarrhoea, lasting a median of two days. Thirty-four (31%) of episodes were also accompanied by vomiting (Table 3). Blood in stool was observed in 17 (17%) episodes while no episodes with rice water stool were observed. Ninety-one (83%) of children did not experience dehydration. Most cases (n = 107, 97%) were treated as outpatients, two (2%) required IV rehydration before discharge, and one (1%) was referred for hospital admission.

**Table 3:** Clinical characteristics of diarrhoea episodes (n = 110).

Characteristics	n (%) or median [IQR]
Duration of diarrhoea (days)	2 [1, 3]
Maximum no. of loose stools in 24 h	2 [2, 3]
Type of diarrhoea *(n = 101)*	
Bloody	17 (17)
Mucoid	34 (34)
Rice water	0 (0)
Watery	46 (46)
Other	4 (4)
Vomiting (% yes)	34 (31)
Maximum no. of vomiting episodes in 24 h	2 [1, 2]
Fever (axillary temperature ≥37.5°C)	18 (16)
General condition on arrival	
Normal /unconscious	98 (89)
Restless/irritable	10 (9)
Lethargic/unconscious	2 (2)
Abdominal pains (% yes)	59 (54)
Ability to feed (% yes)	78 (71)
Normal respirations (% yes)	107 (97)
Skin pinch	
Normal	100 (91)
Slow	9 (8)
Very slow	1 (1)
Tears	
Normal	95 (86)
Decreased	14 (13)
Absent	1 (1)
Hydration status	
No dehydration	91 (83)
Some dehydration	18 (16)
Severe dehydration	1 (1)
Weight-For-Age Z-score *(n = 103)*	-0.61 [-1.31, 0.38]
Visit outcome	
Discharge after IV rehydration	2 (2)
Treated as outpatient	107 (97)
Referred for admission	1 (1)

### Formation of CIDRZ severity scale

Six out of the 11 consolidated items loaded uniquely for severity: maximum number of times vomiting per day, respirations, skin pinch, behavioural signs, tears, and hydration status (Table 4). Severity was the only construct measured by the scale. Hydration status was excluded from the final score due to overlap with component factors of dehydration, such as skin pinch and behavioural signs. Each item was assigned a total number of points out of three based on the seriousness of the symptom. The total points possible for each diarrhoea episode in the final severity scoring scale was 13 points (S1 Table). The overall scale had substantial reliability (Cronbach’s alpha = 0.74).

**Table 4:** Factor extraction with orthogonal rotated factor loadings above 0.40.

Items	Factor 1^a^
Maximum number of times vomiting per day	0.454
Behavioural signs	0.619
Respirations	0.608
Skin pinch	0.487
Tears	0.812
Cronbach alpha = 0.74	

^**a**^Factor 1 can be described as severity.

### Comparison of scores

The median severity scores were as follows: 5 (Vesikari), 2 (Clark), 1 (CODA), and 0 (DHAKA and CIDRZ) (Table 5). The cut-off value for moderate-to-severe diarrhoea for CODA (sensitivity = 100%, specificity = 79%) was ≥ 2. Both Clark (sensitivity = 100%, specificity = 88%) and DHAKA (sensitivity = 100%, specificity = 98%) had a cut-off of ≥ 4. CIDRZ (sensitivity = 100%, specificity = 99%) had a cut-off of ≥ 5. Vesikari (sensitivity = 100%, specificity = 78%) had the highest cut-off points at ≥ 7.

**Table 5:** Summary of scores.

Score	Minimum	Maximum	Median (IQR)	Maximum possible points	Cut-off for severe diarrhoea*
Modified Vesikari	3	10	5 (5, 6)	15	7
Modified Clark	1	7	2 (2, 3)	14	4
Modified CODA	0	5	1 (0, 1)	11	2
DHAKA	0	10	0 (0, 0)	12	4
CIDRZ	0	11	0 (0, 1)	13	5

*The cut-off for each score was the value that maximized the sum of the specificity and sensitivity.

**Fig 1:**
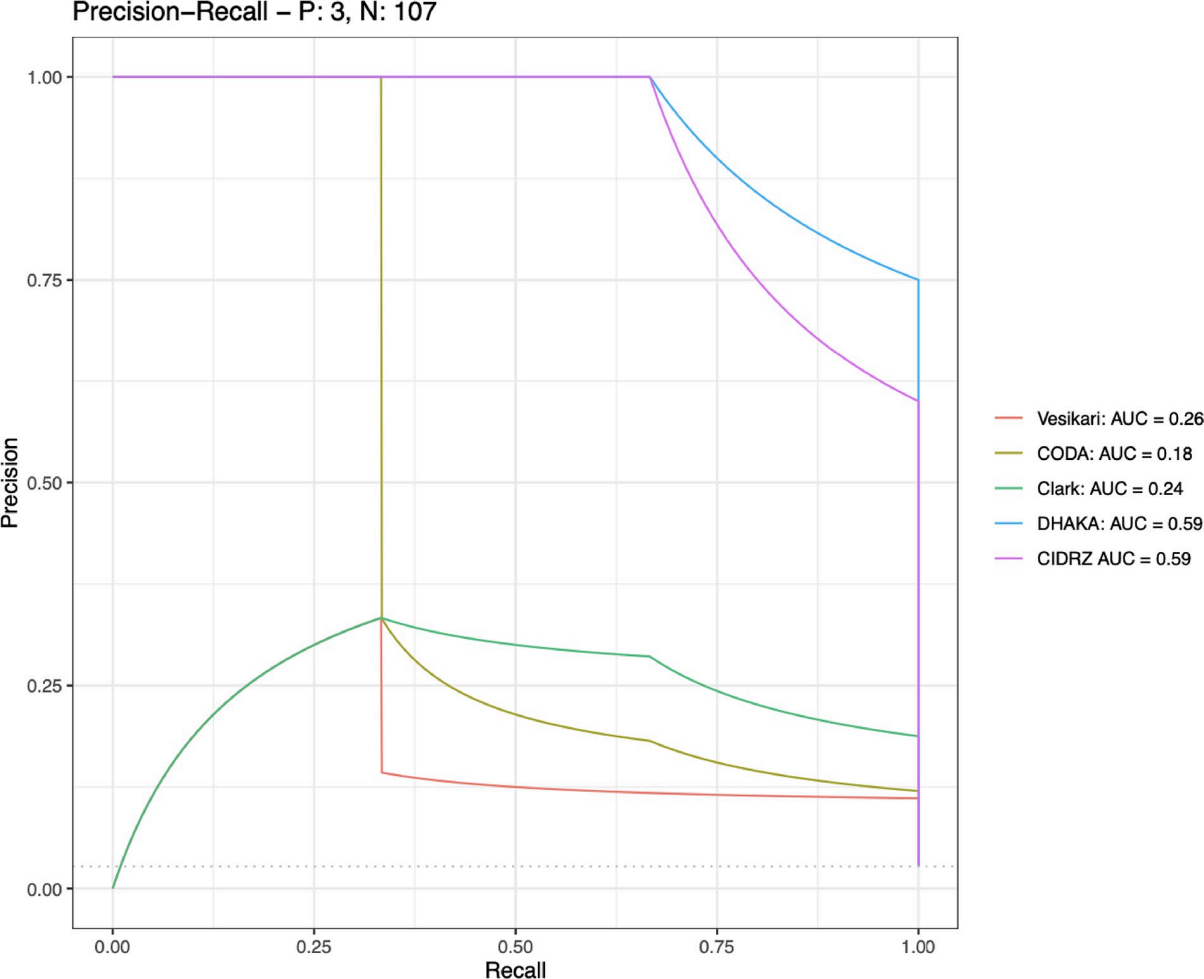
Precision-recall area under the curve (AUC).

The precision-recall AUC of each scoring tool predicting hospitalisation or the necessity of IV rehydration was 0.26 for Vesikari, 0.18 for CODA, 0.24 for Clark, 0.59 for DHAKA, and 0.59 for CIDRZ (Fig 1). Overall, the CODA score had the least agreement with the CIDRZ scale (k: 0.13, 95% CI: -0.13, 0.39), followed by Vesikari (k: 0.20, 95% CI: -0.17, 0.56), Clark (k: 0.20, 95% CI: -0.17, 0.56), and then DHAKA (k: 0.56, 95% CI: 0.11, 1.00).

## Discussion

These data derived from passive health facility-based surveillance of acute gastroenteritis in Zambia underscore differences between diarrhoeal severity scoring instruments and the need to compare different scoring criteria across studies. A CIDRZ severity tool combining clinical characteristics from existing major scoring tools had a higher precision and recall compared to the Vesikari, Clark, and CODA scores in predicting hospitalisation or utilization of intravenous rehydration among children under five (AUC: 0.59). The CIDRZ score performed similarly to the DHAKA (AUC: 0.59) score. The lower performance of some existing scoring tools suggests that they may not be ideally suited for use in the context of outpatient health facilities, especially in resource limited settings.

Our analysis showed that some existing scores had similar recall but lower precision (i.e., lower specificity and a higher false positive rate) compared to the CIDRZ and DHAKA scores. These differences may be attributed to the scores being used outside of the setting and context for which they were constructed; it also underscores important differences between severity scores. The Cohen’s kappa for CIDRZ and CODA test was the lowest of the scores assessed (k: 0.13, 95% CI: -0.13, 0.39) indicating only a slight agreement in classifying severe diarrhoea. CODA was developed to assess long-term negative use in community-based study settings where surveillance is frequent at weekly or bi-weekly intervals, caregivers may not seek clinical care or have access to clinical tools, and fieldworkers rather than trained clinicians are conducting the assessments, which is very unlike clinical trial or healthcare settings. Also, long-term growth and weight changes are the primary outcomes of interest in such studies, which could further explain the lack of agreement [26]. Only DHAKA, which was created for assessing dehydration in children in healthcare settings, demonstrated at least moderate agreement (k: 0.56, 95% CI: 0.11, 1.00) with the CIDRZ tool.

Aetiologic-specific scoring tools may also not be the most appropriate for use in acute primary healthcare settings. The Vesikari (AUC: 0.26) and Clark (AUC: 0.24) scores, which are designed to characterize illness due to rotavirus infection, demonstrated low precision and recall. While the Clark score has been shown to agree poorly with the Vesikari score in previous studies [9,27], the scoring tools performed similarly in this analysis. Though rotavirus is the most frequently isolated pathogen among hospitalised diarrhoea cases, given the changing epidemiology of diarrhoeal disease following the introduction of oral rotavirus vaccines, an aetiologic-specific assessment may be most appropriate in research settings where molecular diagnostics are more commonly used [28–31]. Also, diarrhoea aetiology is not a determining factor in recommending appropriate treatment (except in the case of dysentery), suggesting that such tests may have lower utility when measuring childhood diarrhoeal severity in healthcare settings [4].

Each score has distinct characteristics. The CODA score was the only one to include anorexia, a symptom that can be hard to define. More tangible signs of illness, such as vomiting, were reported more consistently; only the DHAKA score lacked this characteristic. Agreement between the CIDRZ and DHAKA scores was to be expected, given the high number of common variables between them. The CIDRZ score did not weight ‘very slow’ skin pinch or ‘lethargic’ behaviour as highly as the DHAKA score. In addition to variables derived from the DHAKA score, the CIDRZ scale included maximum daily vomiting. However, among our study population the addition of this characteristic did not lead to higher precision and recall.

Though hydration status was a contributing factor to diarrhoea severity based on the EFA, it was excluded from the final CIDRZ scale due to overlap with other score attributes. Skin pinch and behavioural signs, which are included as factors in the CIDRZ scale, are also individual component parts of the WHO ICMI assessment of dehydration. Including hydration status, skin pinch, and behavioural signs would have weighted those components more heavily than others and limited the score performance relative to WHO ICMI. The score reliability with hydration status included (Cronbach’s alpha = 0.79) was similar to the reliability of the final CIDRZ score. We were unable to use percentage difference between weight at enrollment and weight at discharge to measure dehydration because weight at discharge was reported for only 15% of children. Other common measures of dehydration, including urine output and percentage difference between enrollment and discharge weight, should be assessed in future studies [32].

This study was limited by a small sample size and low incidence of severe diarrhoeal disease. In our study, hospitalisation and administration of IV fluids were considered to be proxies for severe diarrhoea. We acknowledge that the decision to administer IV fluids often varies among facilities and can depend on factors other than diarrhoea severity, such as IV fluid availability, hospital capacity, clinical preference, or facility guidelines. Also, modifications were made to the Vesikari, Clark, and CODA scores based on data availability. While this decreased the total points available in each instrument, the modifications were made consistently across scores. The CIDRZ scale also contains a mix of variables that rely on both the caregiver recollection and a clinician’s assessment, which could be valuable. A potential improvement to the score could be to quantify symptoms, where possible, to minimize subjectivity.

Despite these limitations, our analysis confirms that existing diarrhoea severity scores have high recall as predictors of health-seeking behaviours associated with two serious clinical outcomes: hospitalisation and IV rehydration. In healthcare settings in LMICs, DHAKA or the CIDRZ severity scale may predict severe diarrhoeal episodes with more precision. The data used and collected are feasible for use in the context of community health clinics as well as larger hospitals or health facilities. Validating the CIDRZ tool among a larger group of children would increase statistical power to discern whether the addition of maximum vomiting episodes improves predictive ability compared to DHAKA. It is recommended that future studies choose their severity scale carefully while also considering context (community versus healthcare settings) and disease aetiology.

## Supporting information

S1 Table CIDRZ scale. (DOCX)

S1 File (DOCX)
