# Supplementary material for: Development of a diarrhoea severity scoring scale in a passive health facility-based surveillance system
Source: PLoS One. 2022 Aug 15;17(8):e0272981. doi: 10.1371/journal.pone.0272981 (PMC9377573; doi:10.1371/journal.pone.0272981)
Supplement: S1 Table — (DOCX) [file pone.0272981.s001.docx]

| **Component** | **Score** | | | |
| --- | --- | --- | --- | --- |
|  | **0** | **1** | **2** | **3** |
| Max number of times vomiting per day | - | 2-3 | 4-5 | $\geq$6 |
| Behavioural signs | - | - | Restless/ irritable | Lethargic |
| Skin pinch | Normal |  | Slow | Very slow |
| Tears | - | Decreased | Absent | - |
| Respirations | Normal | - | Deep | - |

**S1 Table. CIDRZ Scale**
